# Persistent Coxsackievirus B3 Infection in Pancreatic Ductal Cells *In Vitro* Downregulates Cellular Polyamine Metabolism

**DOI:** 10.1128/msphere.00036-23

**Published:** 2023-04-25

**Authors:** Vincent Mastrodomenico, Natalie J. LoMascolo, Mason R. Firpo, Maria del Mar Villanueva Guzman, Adam Zaporowski, Bryan C. Mounce

**Affiliations:** a Department of Microbiology and Immunology, Loyola University Chicago, Stritch School of Medicine, Maywood, Illinois, USA; b Infectious Disease and Immunology Research Institute, Loyola University Chicago, Stritch School of Medicine, Maywood, Illinois, USA; Wake Forest University

**Keywords:** persistence, coxsackievirus, polyamines, pancreatic ductal cells, enterovirus

## Abstract

Picornaviruses infect a wide variety of cell types *in vitro*, with rapid replication kinetics and pronounced cytopathic effect. Coxsackievirus B3 (CVB3) can also establish a persistent infection *in vivo* that can lead to pathology, including dilated cardiomyopathy and myocarditis. One model system to study persistent infection is the pancreatic ductal cell line PANC-1, which CVB3 infects and is maintained indefinitely. We have characterized this model for CVB3 infection to study persistent infection for over 6 months. We find that CVB3 rapidly replicates within PANC-1 cells without robust cytopathic effect, and after 1 month in culture, titers stabilize. We find that infection does not significantly affect cellular viability. Persistent virus reverts to lytic infection when transferred to Huh7 or Vero cells. We find that persistent CVB3 adapts to PANC-1 cells via mutation of its capsid proteins and, curiously, the viral polymerase (3Dpol) to generate a high-fidelity polymerase. Persistent infection is associated with reduced cleavage of eIF4G, reduced plaque size, and decreasing particle infectivity. We further find that polyamine metabolism is altered in persistently infected cells, with the rate-limiting enzyme ornithine decarboxylase (ODC1) reduced in translation. We further find that targeting polyamine synthesis reduces persistent infection without affecting the viability of the PANC-1 cells. Finally, we find that viral fidelity is essential to maintaining CVB3 infection, and targeting viral fidelity reduces persistent virus infection. Together, these data highlight a novel role for polyamines and fidelity in persistent CVB3 infection and suggest avenues for therapeutic development to target persistent infection.

**IMPORTANCE** Enteroviruses are significant human pathogens that can cause severe disease, including cardiomyopathies. Viruses like coxsackievirus B3 (CVB3) can cause tissue damage by lytically infecting cells; however, CVB3 can also persistently infect, which has been associated with several pathologies. Studying persistent infection *in vitro* is challenging, as CVB3 lytically infects most cellular model systems. Here, we show that CVB3 establishes persistent infection in pancreatic ductal cells *in vitro*, similar to prior studies on other coxsackieviruses. We also show that this infection results in adaptation of the virus to these cells, as well as changes to cellular metabolism of polyamines.

## INTRODUCTION

Enteroviruses are common human pathogens that frequently cause outbreaks, including recent outbreaks of enterovirus D68 (EV-D68) and enterovirus A71 (EV-A71). While typically mild, these and related viruses can cause a range of pathologies, including meningitis and acute flaccid paralysis, for which we have no treatment. Coxsackievirus B3 infection is associated with dilated cardiomyopathy, as it is believed that the virus can persist in cardiac tissue (1). A link between CVB3 infection and diabetes has been suggested (2), though the molecular mechanisms underlying this link remain unknown. Though CVB3 infection is typically acute, diabetes onset associated with CVB3 may be due to chronic inflammation during persistent infection (3). Further, the fundamental mechanisms whereby CVB3 maintains a persistent infection in some tissues (4, 5) and a subset of infections is unclear.

Typical cell culture infection with coxsackievirus results in lytic clearing of a variety of cells, such as the common model systems HeLa, Vero, and 293T. However, coxsackievirus can establish a persistent infection in some cell systems, including human pancreatic ductal carcinoma cells (PANC-1) (6). Prior work showed that coxsackievirus (B1 [7] and B4 [6] serotypes, specifically) can establish persistent infection in PANC-1 cells, with infection maintained for over a year with viral loads maintained at a constant level. Interestingly, significant adaptations occur during this persistent infection, both in the virus (7) and the cell (6, 8 to 10). CVB1 develops mutations in the structural proteins and the 5′ untranslated region (UTR), though the impact of this on infection is unclear (7). Whether other enteroviruses similarly adapt is unexplored.

On the cellular side, PANC-1 cells exhibit significant changes in the transcriptome (9) and proteome (10), namely, in secretory pathways and antiviral responses. The changes in secretory pathways result in an altered secretome that could significantly affect pancreatic cell function and environment. There were also significant changes in cellular metabolism and mitochondrial function (6). Interestingly, changes in pancreatic beta cell survival and differentiation were altered in CVB1-infected cells, though this change appeared to be virus strain-dependent. Given the central role of pancreatic beta cells in maintaining insulin responses and glucose homeostasis, it is vital to understand how infection can change these cells.

We established a persistent CVB3 infection in PANC-1 cells that has been maintained for months with no significant changes in virus titer. We observe viral genetic adaptation to the cells and metabolic changes in the cells, specifically in polyamine homeostasis. We further show that targeting persistent CVB3 infection of PANC-1 cells with known and preclinical antivirals has the potential to clear virus without significant changes in cellular viability. These results extend the persistent models for coxsackievirus infection and demonstrate a link between persistent infection of these cells and polyamine metabolism, which has significant implications for cell viability and function.

## RESULTS

### CVB3 establishes a persistent infection in PANC-1 cells.

**FIG 1:**
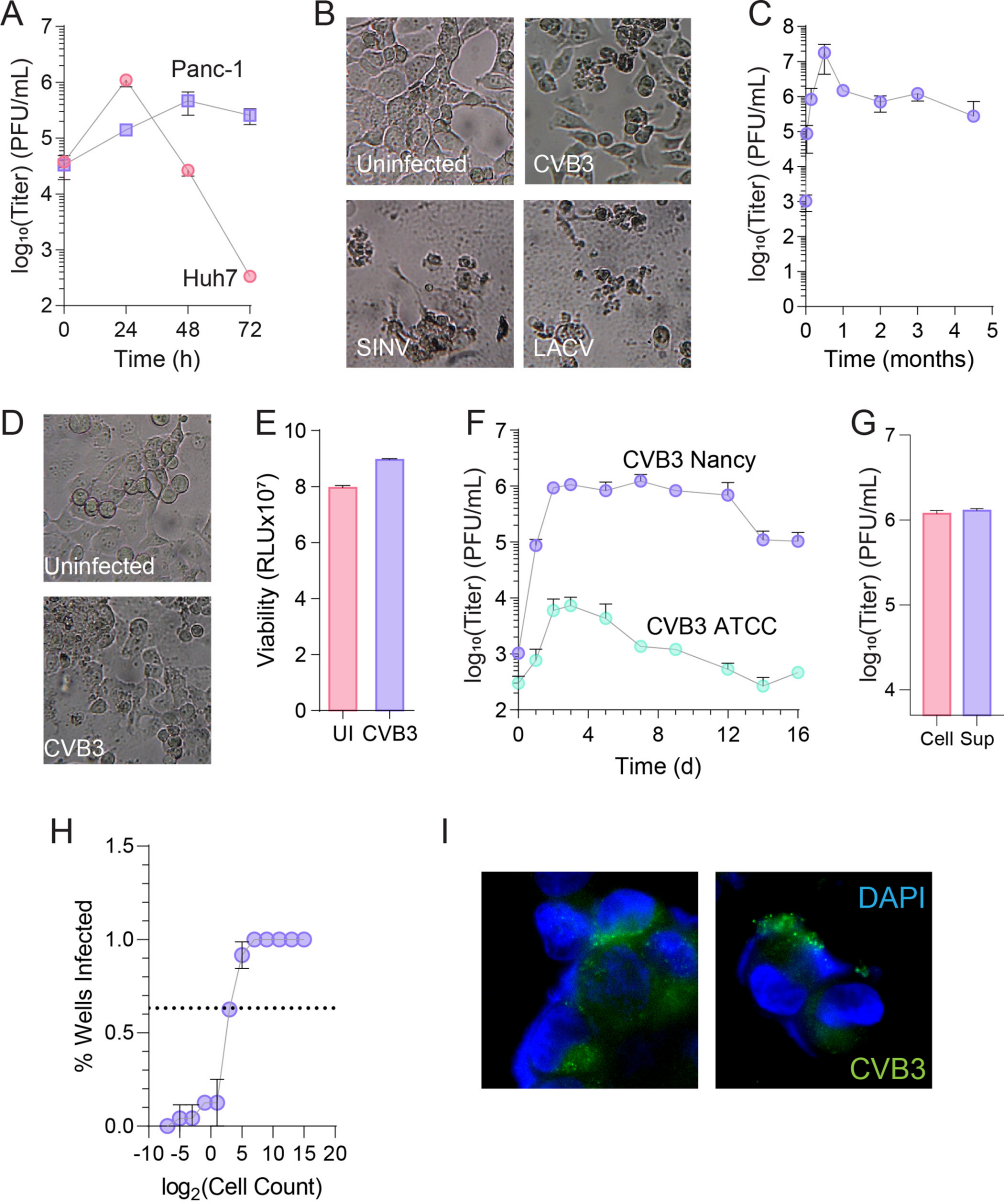
CVB3 established a persistent infection in PANC-1 cells. (A) Huh7 and PANC-1 cells were infected at a multiplicity of infection (MOI) of 1, and viral titers were measured by plaque assay at the indicated times. (B) PANC-1 cells were left uninfected or infected with CVB3, SINV, or LACV at MOI 1 for 48 h. (C) PANC-1 cells were maintained, and viral titers were measured by plaque assay at indicated times. (D) Brightfield images of uninfected and CVB3-infected PANC-1 cells at 4 months. (E) The viability of uninfected and CVB3-infected PANC-1 cells was measured at 4 months. (F) PANC-1 cells were infected with virus derived from the CVB3 Nancy infectious clone or with virus from ATCC (VR-40), and viral titers were determined at the indicated times. (G) Infected PANC-1 cell supernatant (“Sup”) and cell lysate (“Cell”) were assayed by plaque assay for infectious virus. (H) Limiting dilution assay on single-cell suspensions of CVB3-infected PANC-1 cells on Vero cells. Horizontal line crosses at 63.2%. (I) Immunofluorescence for nuclei (DAPI [4′,6-diamidino-2-phenylindole]) and virus (J2, anti-dsRNA) on CVB3-infected PANC-1 cells. Error bars represent one standard error of the mean.

Prior work (6) established that PANC-1 cells can be persistently infected by CVB1 and CVB4, which are closely related to CVB3. To confirm that CVB3 could also infect PANC-1 cells, we first infected the cells, along with Huh7 cells, at a multiplicity of infection of 1. We measured viral titers every 24 h for 72 h. As expected, infection of Huh7 cells resulted in a significant increase in titers at 24 h and a subsequent drop in titer, presumably as virus decayed (Fig. 1A, gray circles). In contrast, infection of PANC-1 cells resulted in a steady increase in viral titers that did not significantly drop off within 72 h (Fig. 1A, purple squares). To confirm that the PANC-1 cells remained live upon infection, we also infected PANC-1 cells with CVB3, the alphavirus Sindbis virus (SINV), or the bunyavirus La Crosse virus (LACV). After 48 h, early in infection when persistence has not been established, we acquired brightfield images of the infected cells (Fig. 1B), and we observed that CVB3-infected cells exhibited distinct morphology from uninfected cells, but the cells remained attached. In contrast, SINV and LACV infection resulted in ablation of the cells, suggesting that persistent infection of these cells was at least partially specific to CVB3.

We maintained the infected PANC-1 cells in tissue culture over several months, titering virus approximately every month to confirm that the cells maintained an infection (Fig. 1C). CVB3 infection was maintained at roughly the same titer for the 5 months assayed. Viral titers rapidly increased in the first 2 weeks and then stabilized at around 10^6^ PFU/mL. We imaged these infected cells compared to PANC-1 cells that had been maintained in tissue culture for approximately the same time. We observed no gross morphological differences that were apparent by brightfield microscopy (Fig. 1D). To confirm that these cells were viable, we performed a viability assay on equal numbers of cells from uninfected and CVB3-infected PANC-1 cells that had been maintained for 4 months. We observed no significant difference in readings in the cells (Fig. 1E), suggesting that infection did not change cellular viability. Finally, we considered an additional CVB3 strain to determine if it also established persistence in PANC-1 cells. Thus, we performed an infection with the ATCC strain (VR-30) of CVB3, which has 10 nucleotide differences compared to our cloned CVB3. We observed that the ATCC (VR-30) strain of CVB3 established persistent infection at a significantly lower titer than our cloned strain of CVB3 (Fig. 1F).

To further characterize our persistently infected cells, we measured intracellular and extracellular virus on cells that had been infected for 4 months. Supernatant was collected, cells washed and lysed, and viral titers were determined by plaque assay. We observed equivalent virus titers in the cells and in the supernatant (Fig. 1G), suggesting that intracellular and extracellular infectious virus levels are similar. We next considered the proportion of cells that are infected. To this end, we performed a limiting dilution assay, in which we trypsinize PANC-1 cells into single-cell suspensions and then plate these cells in octuplicate serial dilution on Vero cells. These cells are allowed to infect for 7 days before fixation and staining with crystal violet. Wells were then scored as live (no CVB3 present; stained with crystal violet) or dead (CVB3 present; no crystal violet stain). We then calculated the proportion of wells that were infected for each dilution. These data were then fit to a logistic curve, and the parameters of the logistic curve were used to calculate the percentage of infected cells using the Poisson distribution (11). From these data (Fig. 1H), we approximated that 17.7% of cells are infected (1 in 5.2 cells). To verify these results, we fixed and stained cells with anti-dsRNA antibody (J2) and counted infected and uninfected cells (Fig. 1I). Using this method, we calculated that 14.4% of cells were infected, closely matching our limiting dilution assay. Overall, these data suggest that a subset of cells are infected.

### CVB3 reverts to acute infection after persistent infection.

**FIG 2:**
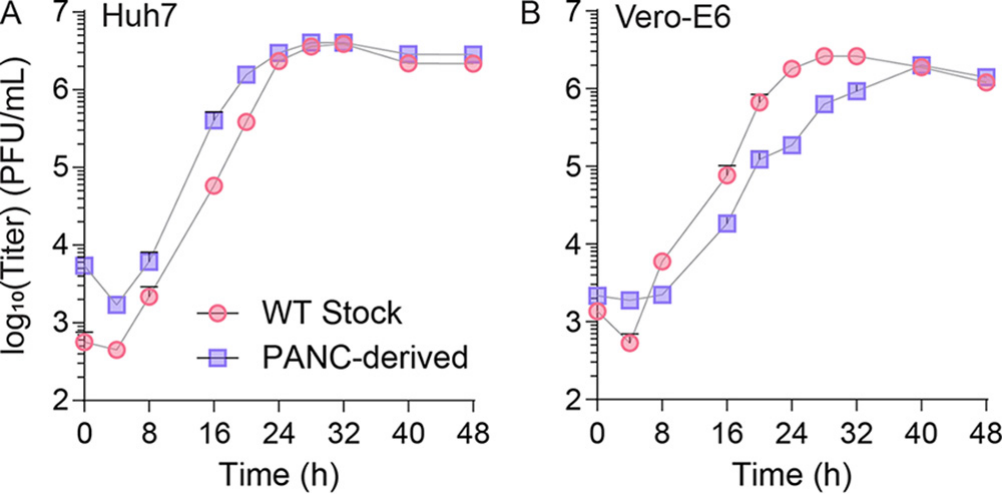
CVB3 reverts to acute infection after persistent infection. (A) Huh7 and (B) Vero-E6 cells were infected with WT CVB3 stock or CVB3 derived from PANC-1 cells after 4 months of persistent infection at MOI 0.01. Viral titers at times indicated were determined by plaque assay. Error bars represent one standard error of the mean.

We hypothesized that CVB3 infection of PANC-1 cells may attenuate the virus as it adapts to persistent infection and that this adaptation may diminish its ability to replicate in other *in vitro* models of infection acutely: Huh7 liver cells and Vero-E6 African green monkey kidney cells. We infected Huh7 (Fig. 2A) and Vero-E6 (Fig. 2B) cells at a multiplicity of infection (MOI) of 0.01 with either CVB3 stock or virus derived from a 4-month infection of PANC-1 cells. Monitoring viral titers over 48 h, we observed no significant differences in viral titers in stock versus PANC-1-derived virus, suggesting that despite being maintained in cell culture for months, the virus maintained its ability to infect both Huh7 and Vero-E6 cells lytically.

### CVB3 adapts to persistent PANC-1 infection.

**FIG 3:**
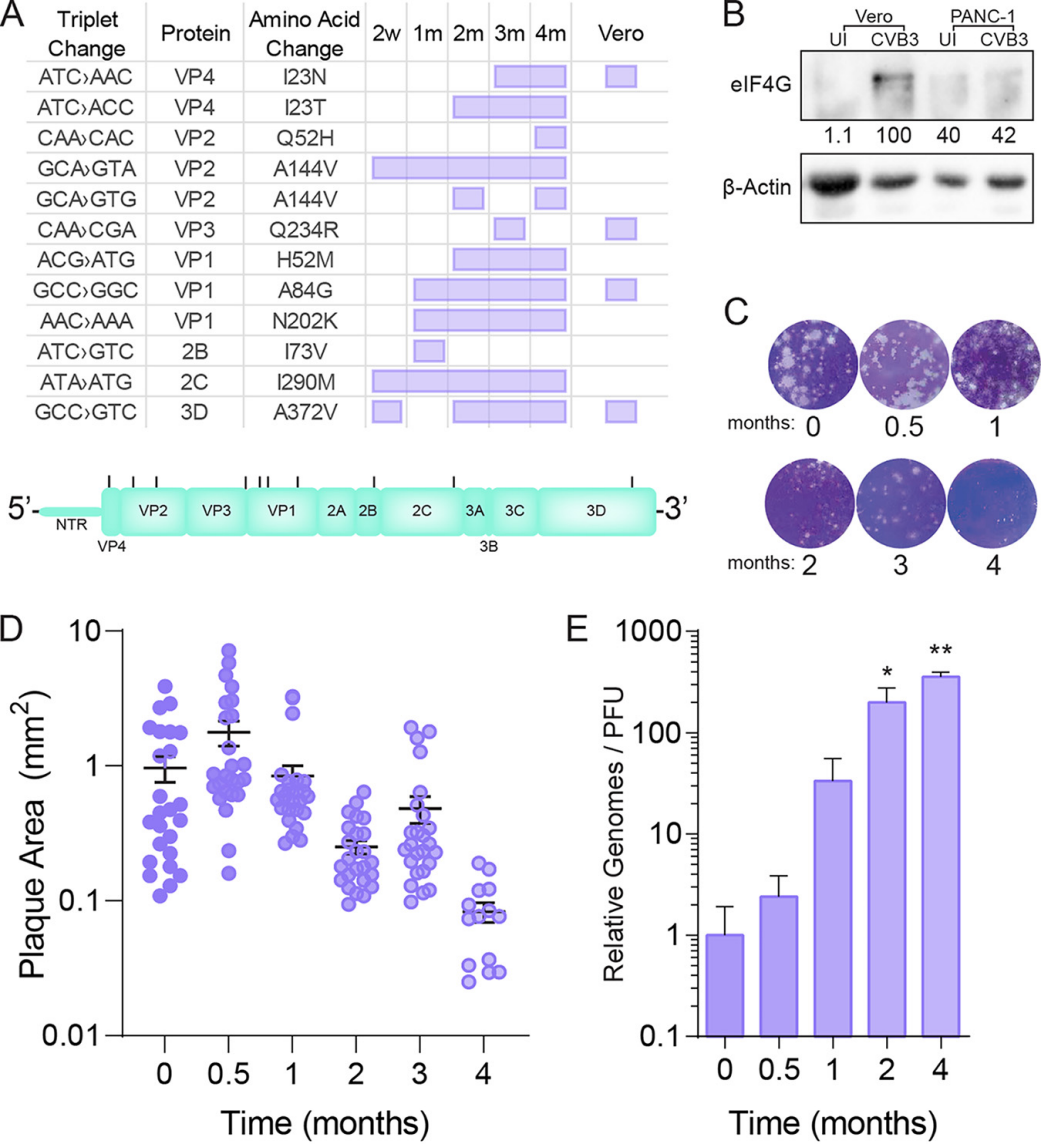
CVB3 adapts to persistent PANC-1 infection. (A) CVB3 derived from a persistent infection in PANC-1 cells was sequenced, and consensus variants were identified. Nucleotide and amino acid changes are indicated, with purple boxes indicating that the mutation was observed in the designated samples. “Vero” column indicates virus passaged for 4 months in PANC-1 cells and subsequently passaged in Vero cells five times prior to sequencing to check for reversion. (B) Vero cells were uninfected (UI) or infected (CVB3) at MOI 1 for 24 h, and PANC-1 cells were uninfected or persistently infected for 4 months. Samples were probed for eIF4G cleavage products. (C) Plaque assay of CVB3 derived from persistent infection of PANC-1 cells over a period of 4 months. (D) Plaque sizes from samples in panel C. (E) CVB3 viral genomes were analyzed by qRT-PCR and compared to viral titer to derive the relative genome/plaque-forming unit (PFU) ratio over the course of 4 months of persistent infection. *, *P* < 0.05; **, *P* < 0.01 compared to 0-month infection by Student’s *t* test. Error bars represent one standard error of the mean.

Having observed no change in CVB3’s ability to undergo lytic replication, we investigated how the virus genetically adapts to PANC-1 persistent infection. To this end, we purified RNA from samples taken every month and reverse-transcribed, amplified, and Sanger sequenced the viral genome. Upon aligning with our stock CVB3 Nancy sequence, we observed several mutations accumulated across the genome (summarized in Fig. 3A). We observed one of the mutations in 3D^pol^, the viral polymerase, at amino acid 372 (3D^A372V^). The mutation has been previously described to reduce viral fidelity (12, 13), preventing the misincorporation of nucleoside analogs. In addition to this mutation, we observed several mutations in the viral structural proteins VP1 to 4. Many of these mutations (VP1^H52M^, VP1^A84G^, VP1^N202K^, VP2^A144V^, and VP4^123T^) were maintained over several months in the samples. We also observed VP3^Q234R^, which we previously described as mediating resistance to polyamine depletion (14). We observed fewer mutations in the nonstructural proteins, 2B^I73V^, 2C^I290M^, and 3D^A372V^. The 2C and 3D mutations were observed over several months of continuous culture. We further determined whether these mutations would revert back to wild-type sequence after five passages in Vero cells (Fig. 1A, “Vero”). We found that the VP1^A84G^, VP3^Q234R^, VP4^I23N^, and 3D^A372V^ mutations were maintained after passage in acute virus infection models. These data could suggest a fitness advantage for these viruses or that these mutations contribute to both acute and persistent infection.

A hallmark of picornavirus infection, including for CVB3, is the cleavage of eIF4G by the 2A viral protease, which limits host translation and promotes translation from the internal ribosomal entry site (IRES) on the viral genome (15, 16). We anticipated that CVB3 may not cleave eIF4G with persistent infection and that cellular viability would be maintained, as we observed (Fig. 1E). First, we infected Vero-E6 cells with CVB3 at MOI 10 for 6 h, where we expected to observe eIF4G cleavage or left the cells uninfected, and we collected equal amounts of PANC-1 cells, maintained in cell culture for 3 months with or without CVB3. We measured the cleavage of eIF4G by Western blot, specifically looking for the cleavage products around 90 kDa. We observed that with CVB3 infection of Vero-E6 cells, eIF4G cleavage was robust, suggesting protease activity as expected during lytic infection (Fig. 3B). In contrast, the PANC-1 cells exhibited little to no eIF4G cleavage, regardless of infection state, indicating that despite being persistently infected, the cells maintained eIF4G. This is despite observing no changes in the viral protease upon viral adaptation to persistent infection (Fig. 3A).

CVB3 produces plaques when infecting Vero-E6 monolayers overlaid with agarose-containing media in approximately 48 h in our assays. To determine if persistent infection of PANC-1 cells resulted in changes in plaque morphology or size, we performed plaque assays with virus isolated immediately after infection, 2 weeks after infection (0.5 months), or 1 to 4 months after infection. We observed that the virus isolated after 4 months exhibited small plaques (Fig. 3C) and required an additional 24 h of incubation to observe countable plaques for a total incubation time of 72 h. Using ImageJ, we measured the plaque sizes after 48 h of incubation and observed a significant reduction in plaque size after approximately 2 months (Fig. 3D). Finally, we extracted virus from samples over the first 4 months of infection and measured viral genomes using CVB3-specific primers. We then compared the relative number of viral genomes to the viral titer to obtain the genome-to-PFU ratio as a measure of particle infectivity. We observed that as persistent infection was maintained, the ratio of viral genomes to infectious virus significantly increased (Fig. 3E), suggesting an increasing particle-to-PFU ratio. Overall, these data suggest that CVB3 adapts to persistent infection of PANC-1 cells, and part of this adaptation results in smaller plaque sizes, perhaps reflected by the mutations observed in sequenced virus (Fig. 3A).

### Persistently infected PANC-1 cells have reduced polyamine metabolism.

**FIG 4:**
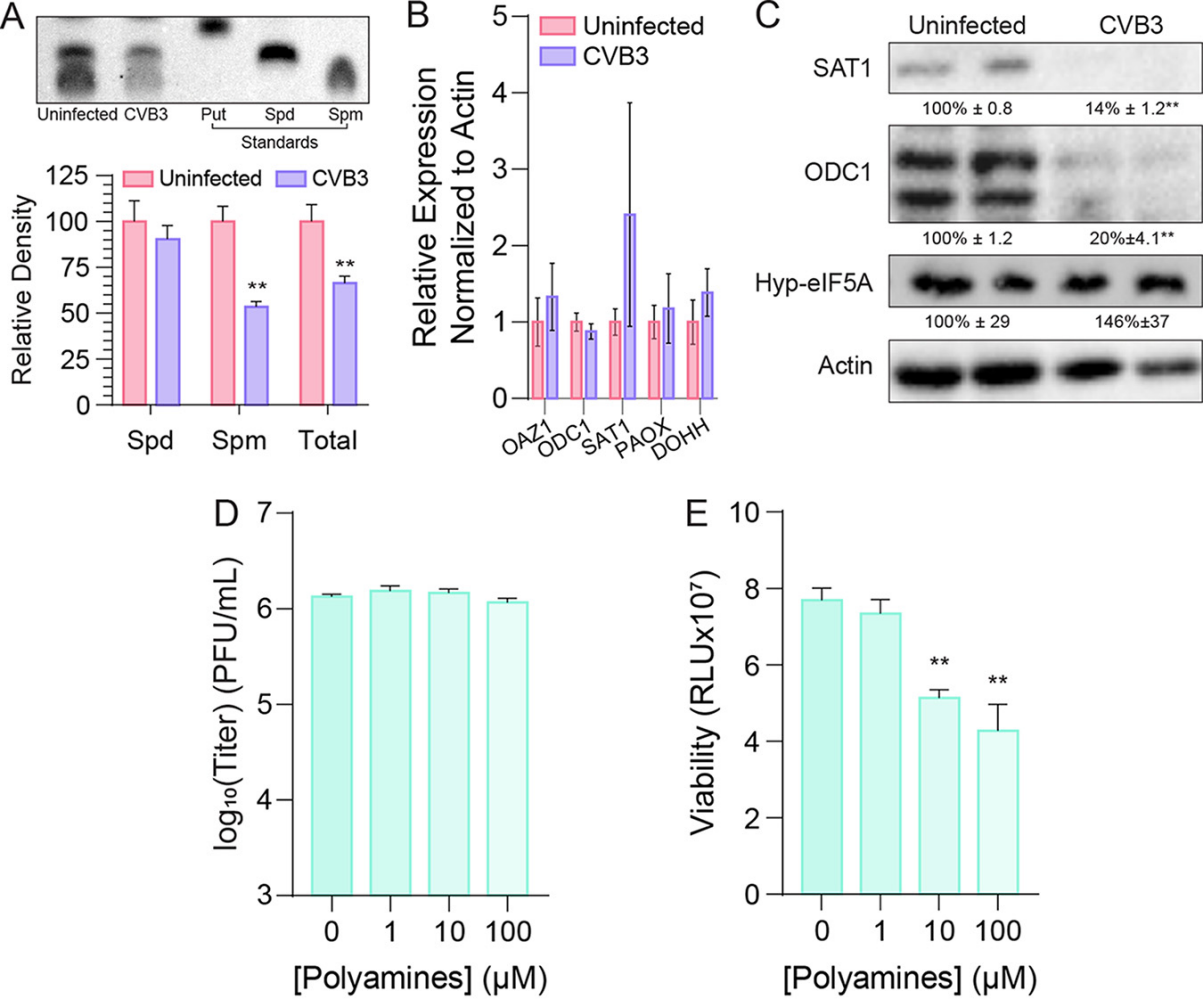
Persistently infected PANC-1 cells have reduced polyamine metabolism. (A) Uninfected and CVB3-infected PANC-1 cells were analyzed by thin-layer chromatography for polyamines (Put, putrescine; Spd, spermidine; Spm, spermine). Bands were quantified by Image J (below). Cells were analyzed by (B) qRT-PCR and (C) Western blot for polyamine synthetic genes. (D) Infected PANC-1 cells were treated with increasing doses of polyamines (equimolar mixture of putrescine, spermidine, and spermine), and viral titers were measured 24 h later. (E) Viability of cells from panel D was measured after 24 h of treatment. **, *P* < 0.01 by Student’s *t* test comparing infected to uninfected (A) or comparing untreated to polyamine treated (E). Error bars represent one standard error of the mean.

Polyamines are critical molecules for both cellular functions (17) and CVB3 replication (14, 18, 19), and we hypothesized that persistent replication might be due to changes in polyamine metabolism, resulting in a detente between the virus and the host. Our observation of the emergence of mutant CVB3 (VP3^Q234R^), resistant to polyamine depletion, further highlighted this potential connection. As a first test, we measured polyamine levels in persistently infected cells versus uninfected PANC-1 cells. We performed thin-layer chromatography (TLC) (20) on cells maintained for 3 months in tissue culture either uninfected or persistently infected with CVB3, normalizing to cell count. In three independent experiments, we observed a significant decrease in signal (Fig. 4A, above), specifically in the band corresponding to spermine. We further quantified (21) the bands corresponding to spermidine and spermine (putrescine was not detectable in our samples) and found a significant decrease in spermine and total polyamine levels but not spermidine (Fig. 4A, below). We tested whether the expression of the genes encoding enzymes in the polyamine synthesis pathway was affected by infection. We observed no significant differences in mRNA levels as measured by qRT-PCR (Fig. 4B). This could be due to two possible effects: the upregulation of SAT1, the acetyltransferase that depletes polyamines, or the downregulation of ODC1, the gatekeeper enzyme responsible for polyamine synthesis. We measured both of these proteins by Western blot. We found that both ODC1 and SAT1 were reduced in abundance in CVB3-infected PANC cells (Fig. 4C). We also measured the levels of eIF5A conjugated to spermidine and hydroxylated to form hypusine-eIF5A (Hyp-eIF5A), which functions in cellular translation (22). We observed no changes in hyp-eIF5A, suggesting that the effects of CVB3 infection of PANC-1 cells were not to reduce the protein levels of polyamine metabolic enzymes globally.

Given that we observed a decrease in cellular ODC1 and polyamines in CVB3-infected PANC-1 cells, we next considered whether supplementing cells with polyamines would enhance viral titers in persistently infected cells. We treated cells with a 1:1:1 mix of putrescine, spermidine, and spermine at 1, 10, and 100 μM for 2 days prior to collecting and titering supernatant. We observed no significant change in viral titers (Fig. 4D), even at the highest supplementation of polyamines. These results align with our prior work that demonstrated that polyamine supplementation does not necessarily enhance CVB3 infection. Conversely, we previously observed that polyamine depletion restricts virus replication, but polyamine supplementation did not increase virus titers (23), suggesting that a low concentration of polyamines is sufficient for virus replication. In the PANC-1 system, we see no change in viral titers, suggesting that virus replication is not altered despite the reduction in cellular polyamines. We next considered whether supplementation of polyamines might change cellular viability and that PANC-1 cells may reduce polyamine levels as a survival mechanism. When we supplemented the cells with polyamines and measured viability, we observed a small but significant reduction in cellular viability (Fig. 4E), suggesting that polyamine levels affect PANC-1 cell survival during CVB3 persistent infection.

We next considered whether these viruses, by their resistance to polyamine depletion, would replicate to higher titers in PANC-1 cells or fail to establish a persistent infection due to overwhelming lytic infection. Previously, we have demonstrated a variety of CVB3 mutations that confer resistance to polyamine depletion in the 2A protease (2A^Q29K^), the 3C protease (3C^Q52R^)17,23, and the structural protein VP3 (VP3^Q234R^)13. We infected PANC-1 cells with these mutant viruses and measured viral titers over several weeks. We observed that each of the mutant viruses successfully established a persistent infection. Although viral titers varied over the time assayed, we did not observe significant differences in the trends for any of the mutant viruses compared to WT CVB3. Thus, mutations conferring resistance to polyamine depletion do not appear to affect viral replication in PANC-1 cells, despite their reduced polyamine levels.

### CVB3 infection can be targeted with antivirals directed against polyamines.

**FIG 5:**
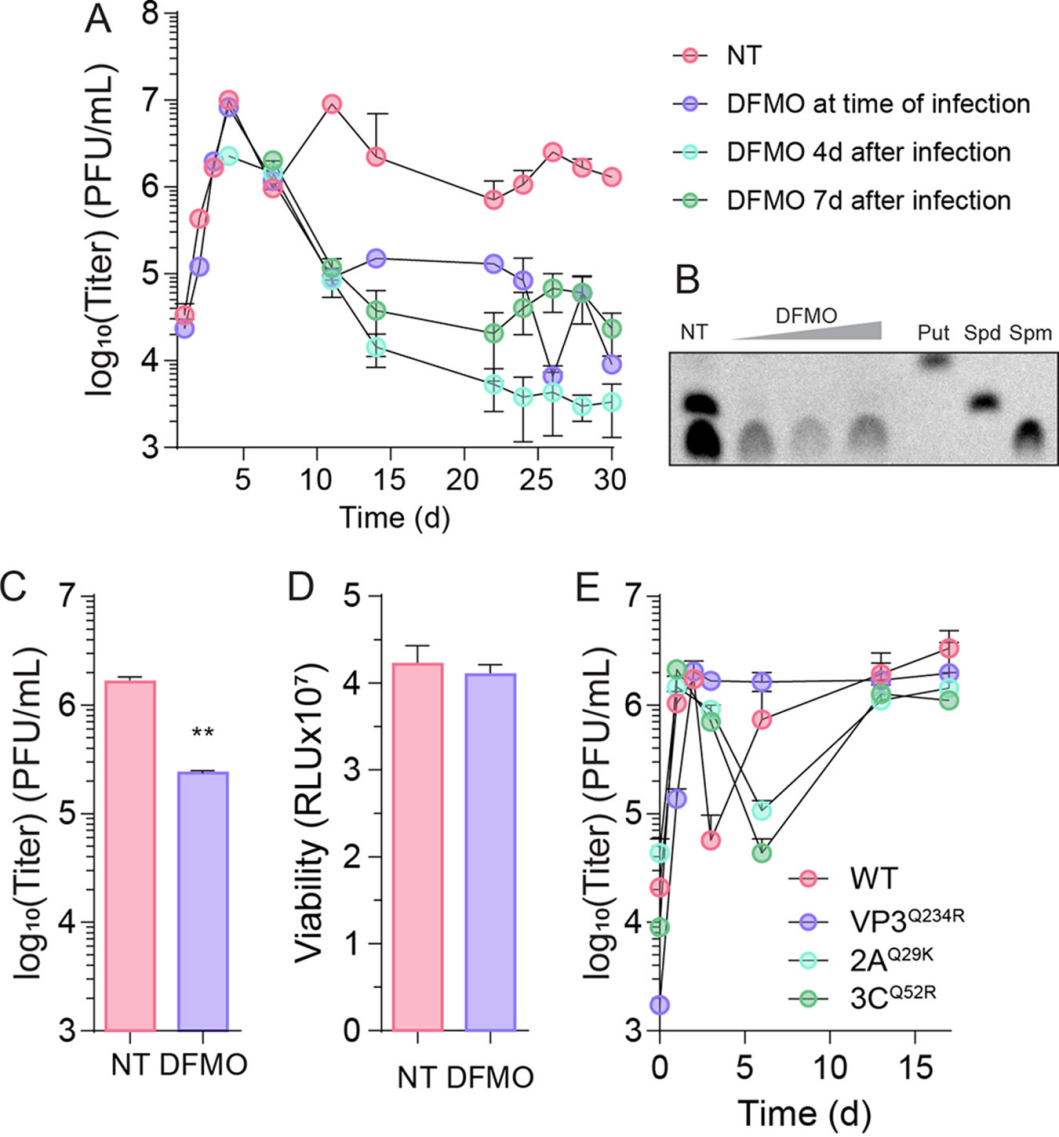
CVB3 infection can be targeted with antivirals directed against polyamines. (A) PANC-1 cells were treated with DFMO at times indicated relative to infection at MOI 0.1 with CVB3. (B) Thin-layer chromatograph for polyamines in PANC-1 cells treated with increasing doses of DFMO (100, 500, and 1000 μM; Put, putrescine; Spd, spermidine; Spm, spermine). Persistently infected PANC-1 cells were treated with 1 mM DFMO, and (C) viral titers and (D) viability were measured 3 days later. (E) PANC-1 cells were infected with WT or mutant (VP3^Q234R^, 2A^Q29K^, and 3C^Q52R^) CVB3, and viral titers were measured at times indicated. **, *P* < 0.01 by Student’s *t* test comparing untreated to DFMO treated. Error bars represent one standard error of the mean.

Our prior work showed that CVB3 replicates in cells with low levels of polyamines, as low as 100 nM (24). Thus, despite reduced polyamine levels in persistently infected cells, we considered whether we could reduce this level further to quench CVB3 replication. To do this, we treated PANC-1 cells with difluoromethylornithine (DFMO), an inhibitor of the rate-limiting enzyme in polyamine synthesis ODC1, from 100 μM to 1 mM. We next infected these cells with CVB3 at an MOI of 0.1 and treated them with DFMO at the time of infection, 4 days after infection, or 7 days after infection. We then measured viral titers by plaque assay over 30 days, maintaining DFMO on the cells throughout that period. We observed that DFMO-treated cells initially produced the same amount of virus as untreated controls (Fig. 5A). However, approximately a week after initiating treatment, we found that DFMO significantly reduced viral titers when added at the time of infection or after infection had been established. We confirmed polyamine depletion by measuring cellular polyamine levels via thin-layer chromatography (Fig. 5B). We observed that DFMO robustly reduced polyamine levels in these cells, though a detectable level of spermine was still present in cells, even at the highest concentration. These data suggest that DFMO can restrict CVB3 replication upon the first days of infection of PANC-1 cells.

To complement these data, we considered whether DFMO could significantly reduce viral titers in PANC-1 cells that had been persistently infected for 3 months. We treated these persistently infected cells with 1 mM DFMO for 7 days and measured viral titers by plaque assay (Fig. 5C). We observed a 10-fold reduction in viral titers in DFMO-treated cells compared to untreated controls. We confirmed that this was not due to cytotoxicity, by measuring cellular viability (Fig. 5D), which was unchanged with DFMO treatment.

We previously characterized viral mutants resistant to DFMO that can replicate in polyamine-depleted cells (14, 18, 25). Since we observe reduced polyamine levels in persistently infected cells, we considered whether these mutant viruses could replicate to higher titers in PANC-1 cells by their ability to replicate robustly in polyamine-depleted cells. We tested three viruses resistant to polyamine depletion, CVB3-VP3^Q234R^ (also observed in our sequencing of persistent virus), -2A^Q29K^, and -3C^Q52R^. All viruses established a persistent infection over 15 days in cell culture (Fig. 5E). However, we noted a distinct drop in viral titers early in infection except with infection with CVB3-VP3^Q234R^. Overall, we observed no significant change in the ability to replicate or establish persistence with these viruses compared to WT CVB3. Together, these data suggest that CVB3 establishes persistent infection in all cells regardless of resistance to polyamine depletion.

### Ribavirin disrupts persistent CVB3 replication but does not clear infection.

**FIG 6:**
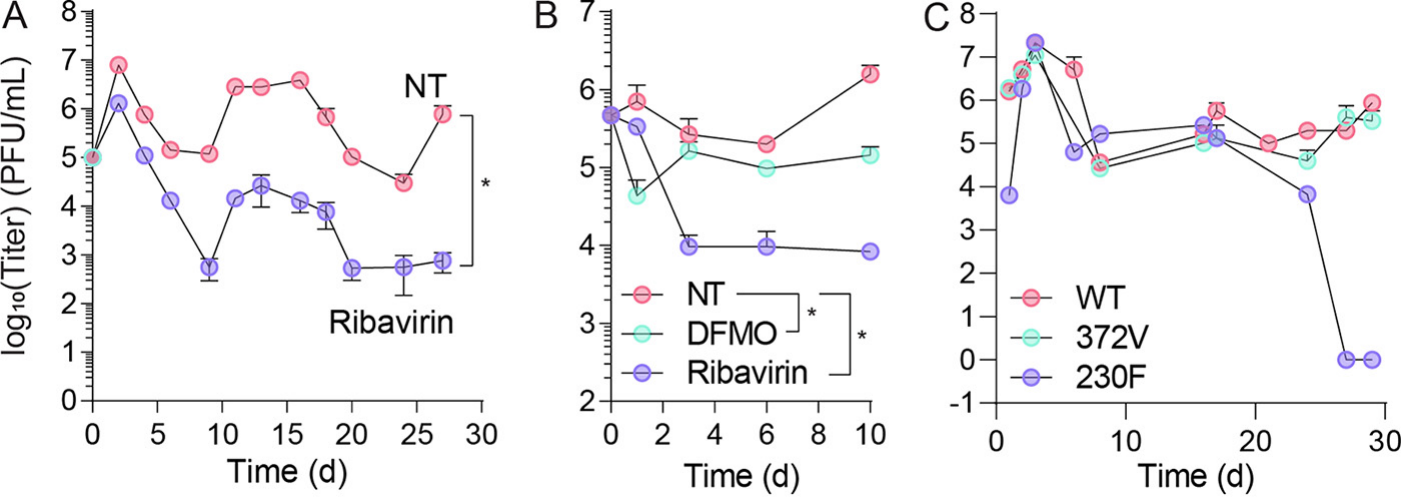
Ribavirin disrupts persistent CVB3 replication but does not clear infection. (A) PANC-1 cells were infected with CVB3 at MOI 0.1 and subsequently treated with 200 μM ribavirin. Viral titers were measured at times indicated by plaque assay. (B) Persistently infected cells were treated with 1 mM DFMO or 200 μM ribavirin, and viral titers were measured at times indicated. (C) PANC-1 cells were infected with WT or mutant (low-fidelity 3D^372V^ and high-fidelity 3D^230F^) CVB3, and viral titers were measured. *, *P* < 0.05 by Student’s *t* test comparing untreated to DFMO treated. Error bars represent one standard error of the mean.

We observed a mutation in 3D^pol^ previously described to reduce CVB3 fidelity and confer resistance to ribavirin (13) (Fig. 3A). Because ribavirin is a clinically relevant antiviral (12), we considered whether ribavirin would reduce CVB3 titers in the PANC-1 persistence model. First, we infected PANC-1 cells with CVB3 and treated with 200 μM ribavirin at the time of infection. We then measured viral titers over 30 days. We observed that ribavirin significantly reduced viral titers, which were maintained for 30 days postinfection (Fig. 6A). Maintaining ribavirin on the cells resulted in a 1,000-fold reduction in viral titers, but the virus was not entirely extinguished, possibly due to the appearance of resistance mutations, such as 3D^A372V^. We next considered whether ribavirin treatment could reduce titers once persistent infection had been established in PANC-1 cells. We used PANC-1 cells that had been persistently infected for 3 months and treated with 200 μM ribavirin over 10 days. We observed that viral titers were reduced by 100-fold over those 10 days of infection, though the virus was not extinguished (Fig. 6B). However, this effect was more pronounced than when cells were treated with 1 mM DFMO.

We observed the emergence of the CVB3-3D^372V^ mutant, which has been demonstrated to have high fidelity and is resistant to ribavirin (13). We considered whether this mutant would affect the establishment of persistent infection. To this end, we infected PANC-1 cells with CVB3-3D^372V^ and measured viral titers over 30 days (Fig. 6C). We observed no change in viral titers compared to WT CVB3. In addition, we infected cells with CVB3-3D^230F^, a polymerase mutant, with reduced fidelity. We observed that this virus initially established a persistent infection that lasted 25 days, but we could not detect virus at 25 days in tissue culture. These results suggest that virus fidelity may play a role in the maintenance of CVB3 persistence and that reducing virus fidelity could reduce persistence.

## DISCUSSION

Understanding CVB3 infection *in vitro* significantly contributes to understanding how CVB3 replicates in the human body. Most cell culture systems typically used with CVB3 infection result in cell death and release of infectious virus, from which we have learned much about virus replication and its interactions with the host cell. Extending our models to *in vitro* persistence models allows us to study CVB3 replication in a novel environment and understand how its persistent infection compares to lytic infection *in vitro*. The PANC-1 cell line represents a tractable model for CVB3 persistence, namely, that CVB3 establishes and maintains a long-term infection, with changes to both the host and virus that likely contribute to maintaining this persistence. Since CVB3 can establish persistence *in vivo* and contributes to pathologies, using these *in vitro* persistence models could lead to a better understanding of persistence *in vivo* and novel means of targeting this persistence to prevent disease.

Previous work with the coxsackievirus persistence model in PANC-1 cells has provided significant insight into how the virus and cell interact during infection. The literature has suggested that infection leads to significant changes in the transcriptome and proteome of the cells (9, 10), as well as extensive changes in the viral genome (7). Interestingly, the changes in CVB1 did not overlap the mutations we observed with CVB3, suggesting differential adaptations to persistent infection. Other work on CVB3 has shown that persistent virus can adapt through deletions in the 5′ terminus of the viral genome (26, 27). Importantly, these mutations are observed in cardiomyocytes, a physiologically relevant site for CVB3 persistence. Thus, it appears likely that virus adapts to different tissues via distinct mechanisms and that this adaptation may also be strain specific.

Enteroviruses are associated with a wide array of pathologies, but CVB3’s association with the development of type I diabetes mellites has received significant attention as a potential driving force in disease development. Murine studies in nonobese diabetic mice have suggested that CVB infection reduces the incidence of type I diabetes; however, in older prediabetic mice, type I diabetes onset occurred more rapidly, which may relate to the ability of CVB to replicate with islets (28). Several hypotheses may explain the relation of CVB with type I diabetes, as recently reviewed (3, 29 to 31), and virus-induced changes in cellular metabolism or alteration of the islet microenvironment may contribute to the acceleration of disease. However, direct virus infection and persistence in the pancreas may not be the only factor promoting disease, as persistence in other tissues could seed infection of the pancreas and contribute to disease less directly.

Polyamines are critical molecules for virus replication, and depleting polyamines reduces virus infection. We observed that CVB3-infected PANC-1 cells exhibited significantly reduced levels of polyamines and that the rate-limiting enzyme ODC1 is significantly reduced in infected cells compared to uninfected controls. Thus, the hypothesis that PANC-1 cells reduce polyamine metabolism to control virus infection is tantalizing. However, these cells must also balance this effect with maintaining cellular functions that rely on polyamines, including cell cycling, signaling, and translation. Thus, the reduction in polyamines and subsequent maintenance of CVB3 at reduced titers may represent a detente between the cell and the virus: the virus induces polyamine depletion (via an unknown mechanism), but the cell maintains a reduced level of polyamines to support cellular function, thus reducing but not eliminating virus replication. However, we observed that supplementing cells with polyamines did not reduce virus replication but modestly reduced cellular viability. Thus, there appears to be a connection between virus replication, cellular viability, and maintaining persistence that is at least partially controlled by polyamines. Future work will be required to identify how this balance is maintained and, importantly, if we can target it to reduce persistence and maintain cellular health.

## MATERIALS AND METHODS

### Cell culture.

Cells were maintained at 37°C in 5% CO_2_ and Dulbecco’s modified Eagle’s medium (DMEM; Life Technologies) with bovine serum and penicillin−streptomycin (Fisher Scientific). Vero cells (BEI Resources) were supplemented with 10% newborn calf serum (GeminiBio). PANC-1 (ATCC) and Huh7 cells, kindly provided by Susan Uprichard, were supplemented with 10% fetal bovine serum (GeminiBio).

### Drug treatments.

Cells were seeded with fresh medium with 2% serum for all drug treatments. Following overnight attachment, cells were treated and incubated at 37°C for times indicated below. Difluoromethylornithine (DFMO; TargetMol) was diluted to a 100-mM solution in sterile water and stored at 4°C. For DFMO, cells were treated and incubated for 96 h at 1 mM. N11-Diethylnorspermine (DENSpm; Santa Cruz Biotechnology) was diluted to 10 mM in sterile water and stored at 4°C. For DENSpm, cells were treated and incubated for 24 h at 100 mM. GC7 (Cayman Chemical) was diluted to a 100-mM solution in sterile water and stored at −20°C. For GC7, cells were treated for 24 h at 500 mM along with 500 μM aminoguanidine (Sigma-Aldrich). Ribavirin (VWR) was diluted to 100 mM in sterile water and stored at −20°C. For ribavirin, cells were treated and incubated for 4 h at 400 mM. Polyamines (Sigma-Aldrich) were added to cells at the time of infection at 10 mM unless otherwise indicated. For infections, all drug treatments were performed at the time of infection unless otherwise indicated. For persistently infected experiments, drugs were continually supplemented onto cells with fresh medium with 2% serum upon collection of viral supernatants.

### Viral infections.

CVB3 (Nancy strain) and CVB3 (ATCC strain) were derived from the first passage of virus in Vero cells (BEI Resources). The VP3^Q234R^, 2A^Q29K^, and 3C^Q52R^ mutant viruses were generated via site-directed mutagenesis of the wild-type CVB3 plasmid as previously described (14, 18). LACV (BEI Resources) was derived from the first passage of virus in Huh7 cells. SINV (24) was derived from the first passage in Vero cells. For all infections, DFMO, DENSpm, GC7, and ribavirin were maintained throughout the infection as designated. Viral stocks were maintained at −80°C. For infection, virus was diluted in serum-free DMEM for a multiplicity of infection (MOI) of 0.1 on PANC-1 cells unless otherwise indicated. The viral inoculum was overlain on cells for 10 to 30 min, and the cells were washed with PBS before replenishment of media. Supernatants were collected from CVB3, mutant CVB3 viruses, LACV, and SINV, as indicated.

### Establishment and maintenance of persistently infected PANC-1 cells.

PANC-1 cells (ATCC) were infected with CVB3 (Nancy Strain) at an MOI of 0.1. Cells were maintained in DMEM supplemented with 10% FBS at 37°C in 5% CO2. Medium was collected, and fresh medium was supplemented onto cells weekly.

### Enumeration of viral titers and plaque size measurement.

Dilutions of supernatant were prepared in serum-free DMEM and used to inoculate confluent monolayers of Vero cells for 10 to 15 min at 37°C. Cells were overlaid with 0.8% agarose in DMEM containing 2% NBCS. CVB3 and mutant virus samples were incubated for 2 days, SINV samples were incubated for 3 days, and LACV samples were incubated for 4 days at 37°C. Following appropriate incubation, cells were fixed with 4% formalin and revealed with crystal violet solution (10% crystal violet; Sigma-Aldrich). Plaques were enumerated and used to back-calculate the number of PFU per milliliter of collected volume. Plates were scanned with Epson Perfection 4990 Photo scanner, and plaque sizes were determined using ImageJ software. To measure intracellular versus extracellular virus, cellular supernatant was collected for plaque assay, and cells were thrice washed with PBS. Cells were then overlaid with 500 μL PBS and frozen and thawed three times to release intracellular virus.

### Limiting dilution assay.

PANC-1 cells were trypsinized and inspected for full dissociation into single cells. Cells were added to Vero cells in 96-well plates at a density of 33,000 cells/well in eight replicates. Fourfold serial dilutions were similarly added to Vero cells. The cells were coincubated for 5 days to allow for virus in PANC-1 cells to infect and lyse Vero cells. Cells were subsequently fixed and stained with crystal violet, and wells were scored as either live (stained with crystal violet, intact Veros) or dead (clear well, lysed Veros). The proportion of wells that were dead (infected) was calculated and graphed. Data were fit to a logistic curve, and the intersection of this logistic curve (GraphPad Prism) with a proportion of 63.2% was used to determine the approximate proportion of infected cells (11).

### Cell imaging.

PANC-1 cells were seeded in a 6-well plate at 6 × 10^^5^ cells per well. Following overnight attachment, the cells were infected with CVB3, LACV, and SINV at an MOI of 1 or left uninfected for 72 h. Pictures of the cells were taken with a Levenhuk M500 Base camera with LevenhukLite software (Version ×64). Persistently infected PANC-1 cells were plated at the same density, and photos were taken as previously described. For immunofluorescence images, PANC-1 cells were seeded to coverslips and fixed 24 h later. Fixed cells were then stained for nuclei (DAPI) and dsRNA (J2, Invitrogen) as previously described (32). They were imaged on Zeiss Axio Observer 7 with Lumencor Spectra X LED light system and a Hamamatsu Flash 4 camera using Zen Blue software with a 40× objective.

### Cellular viability assay.

PANC-1 and persistently infected PANC-1 cells were seeded in a 96-well plate at 1.5 × 10^^4^ cells per well. Following overnight attachment, cells were treated with DFMO or polyamines, or infected with CVB3, LACV, and SINV, as previously described above. After the allotted time for drug treatment and infection, 20 mL of CytoTox-Fluor cytotoxicity assay reagent (Promega) was added to each well and incubated in the dark at 37°C for 1 h. The fluorescence of the plate was taken using a SpectraMax iD3 fluorometer (485 nm excitation/525 nm emission).

### Thin-layer chromatography determination of polyamines.

Polyamines were separated by thin-layer chromatography as previously described (23). For all samples, cells were trypsinized (Zymo Research), reseeded with new medium supplemented with 2% serum, collected, and centrifuged. The pellets were washed with PBS and resuspended in 100 μL of 2% perchloric acid. Samples were then incubated overnight at 4°C. A volume of 100 μL of the supernatant was combined with 200 μL of 5 mg/mL dansyl chloride (Sigma-Aldrich) in acetone (Sigma-Aldrich) and 100 μL of saturated sodium bicarbonate (Sigma-Aldrich). Samples were incubated in the dark overnight at room temperature. Excess dansyl chloride was cleared by incubating the reaction with 100 μL of 150 mg/mL proline (Sigma-Aldrich). Dansylated polyamines were extracted with 50 μL of toluene (Sigma-Aldrich) and centrifuged. A volume of 5 μL of the sample was added in small spots to the TLC plate (silica gel matrix; Sigma-Aldrich) and exposed to ascending chromatography with 1:1 cyclohexane: ethyl acetate. The plate was dried and visualized via exposure to UV. Images were quantified by ImageJ.

### Western blot analysis.

Samples were collected using 1 × SDS buffer and run on 10% polyacrylamide gels. Gels were transferred in the Trans-Blot Turbo transfer system (Bio-Rad). Membranes were blocked using 5% BSA in 1× tris-buffered saline with 0.2% Tween (TBST) and probed with primary eIF4G (1:1,000; Santa Cruz Biotech), ODC1 (1:1,000; Invitrogen), SAT1 (1:1,000; Novus Biologicals), and b-actin (1:1,000; ProteinTech) antibody. Membranes were washed in 1× TBST and placed in secondary anti-mouse IgG (1:15,000; Jackson Immunoresearch) or anti-rabbit IgG antibody (1:15,000; Jackson Immunoresearch) and incubated at room temperature for 1h. Again, membranes were washed in 1× TBST, and SuperSignal West Pico Plus chemiluminescent substrate (Thermo-Fisher) was applied to membranes and developed on a molecular imager, Bio-Rad GelDoc XR1 imaging system (Bio-Rad). Images were quantified with ImageJ.

### RNA purification and cDNA synthesis.

The medium was cleared from cells, and TRIzol reagent (Zymo Research) was directly added. The lysate was then collected, and RNA was purified according to the manufacturer’s protocol utilizing the Direct-zol RNA Miniprep Plus kit (Zymo Research). According to the manufacturer's protocol, the purified RNA was subsequently used for cDNA synthesis using high-capacity cDNA reverse transcription kits (Thermo-Fisher) with 10 to 100 ng of RNA and random hexamer primers.

### Viral genome quantification.

Following cDNA synthesis, qRT-PCR was performed using the QuantStudio3 (Applied Biosystems by Thermo Fisher) and SYBR green master mix (DotScientific). Relative gene expression was calculated using the DC_T_ method, normalized to b-actin. Primers (Table 1) were verified for linearity using 8-fold serially diluted cDNA and checked for specificity via melt curve analysis.

**TABLE 1:** qPCR primers used in this study

Primer	Forward	Reverse
β-Actin	5′-CAC-TCT-TCC-AGC-CTT-CCT-TC-3′	5′-GTA-CAG-GTC-TTT-GCG-GAT-GT-3′
OAZ1	5′-GAG-GGA-ATA-GTC-AGA-GGG-ATC-A-3′	5′-GTC-TTG-TCG-TTG-GAC-GTT-AGT-3′
ODC1	5′-AGG-CCG-ACG-ATC-TAC-TAT-GT-3′	5′-GGC-ATC-CTG-TTC-CTC-TAC-TTC-3′
SAT1	5′-GAA-GAG-GTG-CTT-CTG-ATC-TGT-C-3′	5′-CTC-ACT-CCT-CTG-TTG-CCA-TTT-3′
PAOX	5′-GGG-AGT-ACC-TCA-AGA-AGG-AGA-T-3′	5′-CCA-GGT-TGA-AGA-AGG-AGT-TCA-G-3′
DOHH	5′-AGG-CCT-TCG-ATG-ACG-ATT-C-3′	5′-CTC-CTG-ACG-GGT-GTC-TTG-3′

### Sequencing and alignment.

Persistently infected PANC-1 supernatant was collected, and inoculated on Vero cells for 48 h. Supernatant was then collected, and viral RNA was purified, reverse transcribed, and amplified via 2× Platinum SuperFi II Green PCR Master Mix (Invitrogen) using CVB3-specific primers (Table 2). PCR products were cleaned with Zymoclean Gel DNA Recovery (Zymo Research) and sent for Sanger sequencing (Genewiz from Azenta Life Sciences). Sequences were aligned to CVB3 parental genome, and mutants were confirmed by manual chromatogram inspection. For reversion analysis, Vero cells were serially infected with 10 μL of virus inoculum for five passages. After passage, virus was collected and analyzed for mutation as described above.

**TABLE 2:** Sequencing primers used in this study

Primer	Sequence
CVB3 120F	5′-ACCCCCTCCCCCAACTGTAA-3′
CVB3 2339R	5′-GCTGGGACCACTATGTTlTGT-3′
CVB3 2227F	5′-GTGCTGTGTATACCCTGGATAAG-3′
CVB3 3206R	5′-CAGAGTCTAGGTGGTCTAGGTATC-3′
CVB3 3128F	5′-GGAAGCACGGGTCCAATAAA-3′
CVB3 4687R	5′-ACGGTGAGGTGAACAGAATG-3′
CVB3 4574F	5′-GCCAGAATCCTGATGGGAAA-3′
CVB3 5724R	5′-GTATTCTGTGACCTGTCCTACTG-3′
CVB3 5126F	5′-TGCGGACCTGCTCAAATC-3′
CVB3 6515R	5′-CCTGGGTTTAGGTGGAAAGT-3′
CVB3 6398F	5′-CCATAGAGAAGGTAGCGAAAGG-3′
CVB3 3′end R	5′-GCGGAGAATTTACCCCTACTG-3′

### Statistical analysis.

Prism 9 (GraphPad) was utilized to generate graphs and perform statistical analysis. For all analyses, analysis of variance (ANOVA) and two-tailed Student's *t* test was used to compare groups, unless otherwise noted with a value of 0.05.
